# Endoplasmic Reticulum Is Involved in Myocardial Injury in a Miniature Swine Model of Coronary Artery Stenosis Exposed to Acceleration-Associated Stress

**DOI:** 10.1371/journal.pone.0132654

**Published:** 2015-07-13

**Authors:** Haitao Zhang, Meng Chai, Chaozhong Liu, Jinjin Sun, Congchun Huang, Xinya Yu, Yi Tian, Huilan Luo

**Affiliations:** 1 Department of Cardiology, General Hospital of Air Force, PLA, Beijing 100142, China; 2 Department of Cardiology, Beijing Anzhen Hospital, Capital Medical University, Beijing,Institute of Heart Lung and Blood Vessel Disease, Ministry of Education, Beijing 100029, China; 3 General Hospital of Air Force, PLA, Beijing 100142, China; 4 Animal Experimental Center of Fuwai Hospital, National Heart Center of China, Beijing, 100037, China; University of Colorado Denver, UNITED STATES

## Abstract

This study aimed to investigate the effects of myocardial injury in a minimally-invasive miniature swine model with different levels of coronary artery stenosis (CAS) and exposed to maximal tolerated +Gz. Proximal left anterior descending branch was ligated in 20 swine. Five swine underwent a sham operation. A trapezoid acceleration curve was used for +Gz stress. Pathological changes of myocardial tissue were detected by H&E staining. Apoptotic cardiomyocytes were detected by TUNEL. GRP78 and CHOP were investigated by immunohistochemistry and western blot. CAS models were successful in 18 animals.Compared with the sham-operated group (+8.00±0.71 Gz), the maximal tolerated +Gz values of the moderate stenosis (+6.00±0.89 Gz, P<0.05) and severe stenosis groups (+5.20±0.84 Gz, P<0.05) were decreased.Compared with sham animals (12.16±1.25%), after exposure to maximum +Gz, apoptotic cells of the moderate (43.53±8.42%, P<0.05) and severe stenosis group (60.50±9.35%, P<0.05) were increased, MDA content was increased (1.89 and 4.91 folds, respectively, P<0.05), and SOD activity was reduced (-13.66% and -21.71%, respectively). After exposure to maximum +Gz, GRP78 protein expression was low in the sham-operated (0.29±0.05) and mild stenosis groups (0.35±0.04), while expression was high in the moderate (0.72±0.04, P<0.05) and severe stenosis groups (0.65±0.07, P<0.05). CHOP protein expression was not observed in the sham-operated group, while expression was high in the moderate and severe stenosis groups. These results indicated that Under maximum exposure to +Gz stress, different levels of CAS led to different levels of myocardial injury. Endoplasmic reticulum response is involved in the apoptosis of cardiomyocytes after +Gz stress.

## Introduction

In the search to gain advantages in planes combat, higher performance aircrafts are being developed, imposing ever greater acceleration forces on pilots [1]. During flight, pilots suffer from detrimental environmental conditions, such as acceleration-associated stress (+Gz stress), noise, radiations, heat, hypoxia and vibration, all of which can induce cardiac structural damages [2–4], and increasing the risk of coronary heart diseases (CHDs) [5]. Indeed, CHD may occur in pilots 3–15 years younger than in the general population [6,7]. Repeated exposured to high +Gz induce significant physiological adaptation reactions associated with blood pressure regulation (blood volume reduction, cardiac output decreases)[8–11]. In addition, due to mechanical force and severe hemodynamic changes, cardiomyocytes and muscle fibers will suffer from some damages. Some authors have confirmed the effects of high +Gz were similar to hemodynamic changes observed in ischemia reperfusion (I/R) [12].

Oxidative stress is defined as an imbalance between reactive oxygen species (ROS) production and removal [13]. This imbalance causes contractile dysfunction and structural damage to the myocardium, leading to apoptosis [14]. There is increasing evidence that oxidative stress constitutes the basic pathophysiological process involved in I/R injury [15].

Endoplasmic reticulum (ER) stress (ERS)-initiated apoptotic pathway is a self-protecting mechanism aiming to protect the ER homeostasis. The unfolded protein response is an integrated response that aims to restore ER homeostasis by increasing the capacity of the ER to fold and process proteins, and to reduce the protein load in the ER [16]. However, the exact ER response to +Gz is unknown.

In the present study, we used a swine model of coronary artery stenosis (CAS) achieved using a minimally-invasive approach and allowing a precise control of the narrowing degree. Animals were exposed to +Gz stress soon after surgery. The aim of the present study was to assess the maximal tolerated +Gz stress in relation to CAS degree, and to characterize the degree of cardiomyocyte apoptosis induced by ERS.

## Materials and Methods

### Animals

Twenty-five healthy, mature, male Bama miniature swine (mean weight of 23±2 kg, and 10.0±1.6 months old) were purchased from the Heilongjiang Shuangyashan Miniature Swine Farm. Swine were raised in the Animal Center of the Fuwai Hospital for Cardiovascular Diseases, Chinese Academy of Medical Sciences (under the license: SYXK (Beijing) 2008–0016). Animals were fed twice daily with grain according to the animals’ growth requirements using a normal diet, and were allowed free access to drinking water. Their general state, activity, gait, secretions, food and water uptake, urine, stool and body weight were recorded daily before surgery.

The animals used in this study were obtained, cared for, and used in accordance with the Animal Welfare Act and the “Guide for the Care and Use of Laboratory Animals” from the Institute of Laboratory Animal Resources. This study has been approved by the Animal Welfare and Ethics Committee of Fuwai Hospital for Cardiovascular Diseases, Chinese Academy of Medical Science (license #2010-1-20-125ZD).

### Coronary artery stenosis model by thoracoscopy

After a 24-h fast, swine older than 10 months were anesthetized with intramuscular ketamine (35 mg/kg) and diazepam (1 mg/kg) and placed in a right lateral position. Breathing was supported using a Savina intensive care breathing machine (Dräger, Lubeck, Germany). Breathing was observed by an anesthesiologist and the swine was injected with pentobarbital, according to practical requirements. ECG was monitored (Hewlett-Packard, Palo Alto, California, USA). All surgical procedures were performed by a trained surgeon. A 2-cm main operation hole was made on the 4th rib line at the left armpit level, and a thoracoscope was inserted in the left sternal 2 level. A 1-cm auxiliary operation hole was made on the left side of clavicle midline between the 4th rib line. Two vessel forceps were inserted into each hole to push the lung lobes. Pericardium was cut 2 cm from the diaphragmatic nerve, exposing the heart. The most bulky vessel on the heart surface, the left anterior descending (LAD) artery, and its forward diagonal and ventricular branches were identified. Using forceps, the LAD artery was gently separated in its proximal diagonal branch, 1 cm apart from the left main branch. At the proximal LAD artery bifurcation, casing needles of different sizes were placed and the blood vessels were ligated with silk suture. The casing needles were gently withdrawn, thus controlling the degree of narrowing. Following the surgical procedure, each swine underwent a quantitative computerized angiography (QCA) for confirmation of the degree of stenosis using an OEC9800 digital subtraction angiography machine (GE Healthcare, Waukesha, Wisconsin, USA) [17].Using the surgical method of femoral artery exposure, a 6F sheath with a 5F JR4.0 diagnosis catheter (Medtronic, Fridley, Minnesota, USA) was placed into the left coronary sinus. Iodine contrast agent was injected. Using conventional projection angles, the continuous movie method was used to observe the degree of LAD artery stenosis. QCA was performed by the same group of three experienced professional cardiac interventional physicians using the VisionRis quantitative analysis software (Beijing Weiye Future Technology Co., Ltd., Beijing, China) [18]. Vessel diameter of the target lesion and reference vascular diameter were then collected.

The reference vascular diameter was the diameter of the normal vessel proximal to the lesion. The percentage of stenosis was calculated as [1-(vessel diameter of the target lesion/reference vascular diameter)]×100%. Swine were divided into groups according to their CAS degree: mild (stenosis of 20–50% of the reference vascular diameter), moderate (stenosis of 51–70%), and severe (stenosis of 71–90%) [19]. Chest was then sutured and the animals were allowed to recover under standard antibiotic therapy with benzathine penicillin (1.2 million IU/day) by intramuscular injection. Five swine underwent a sham surgery using identical anesthetic and surgical procedures, but without LAD artery ligation. Animals were kept for 1 week under standard diet and received low molecular heparin calcium (2100 IU) 6 h after surgery and then every 12 h, clopidogrel (75 mg/day), aspirin (100 mg/day) and penicillin (4.8 million units/day for 3 days). Their general state, activity, gait, secretions, food and water uptake, urine, stool and body weight were recorded dailyafter surgery. Then, +Gz stress exposure was performed in each group and venous blood (20 mL/sampling) was collected before/after exposure.

### Grouping, +Gz stress exposure and specimen collection

The 23 miniature swine model were divided into sham-operated group (n = 5), mild stenosis group (n = 7), moderate stenosis group (n = 6), and severe stenosis group (n = 5).

The Type 98 centrifuge (China Astronaut Research Training Center Development) is equipped with an 8-meter arm, and can produce a G force ranging from 1.41 to 16 G, with a maximum rate of 6 G/s. The single-pod uniaxial cabin has an effective carrying capacity of 165 kg.

For all centrifuge procedures, swine were placed in a form-fitted aluminum and fiberglass restraint system (barrel with a soft cushion filling) (80 cm tall and 35 cm in diameter) and held in place with straps behind the head, at mid-chest, and across the hips. The restraint system positioned the swine in a normal standing position with the weight of the animal supported along the ventral surface. The head was facing the centrifuge rotation axis. Therefore, the inertial load (+Gz stress) was parallel to the spine in a head-to-buttocks direction, similar to the +Gz stress exposure of pilots of high-performance aircrafts.

Before being placed in the restraint chair, swine were anesthetized with ketamine (10 mg/kg) and diazepam (1 mg/kg) by intramuscular injection. Then, 5-lead (chest) ECG leads were placed. Swine were then exposed to +Gz stress. They first experienced a +3 Gz stress for 10 s, and a trapezoid acceleration curve was used after 5–10 min. Beginning with an initial +3Gz stress exposure, the G-value was increased at a rate of 1 G/s, for 60 s, for each exposure. During the +3 to +9 Gz exposure, if the ECG monitor displayed risk signals (the same lead showing successive premature ventricular contraction three times or more, or ventricular fibrillation), the +Gz stress exposure was immediately stopped. Intervals between exposures were 10 min, and the maximal +Gz stress was +9 Gz. ECG and physiological parameters were continuously recorded using a FE-30 magnetic recorder (Sony, Tokyo, Japan).

Thirty minutes after the centrifuge test, the swine were euthanized by injecting 15% potassium chloride into the superior vena cava. Their chest was opened quickly. The hearts were taken out, flushed with 0.9% saline repeatedly, and kept at -80°C.

### H&E staining of paraffin-embedded myocardial tissues

Heart specimens of miniature swine were rinsed with 0.9% saline. The left ventricle was separated along the interventricular septum. Left ventricular anterior wall (LVAW) tissue was cut into 1×0.5×0.2-cm pieces. Specimens were fixed in 4% paraformaldehyde, dehydrated, embedded in paraffin, sectioned, and stained with H&E. Myocardial tissue pathological changes were observed under an optical microscope.

### Malondialdehyde content and superoxide dismutase activity

Malondialdehyde (MDA) content and superoxide dismutase (SOD) activity was determined in myocardial tissue by the glucosinolates barbituric acid method and by a spectrophotometer method, respectively, using commercial kits (Nanjing Jiancheng Biotech, Nanjing, China) and according to the manufacturer's instructions.

### Myocardial cell apoptosis

An area under the ligature of the LVAW (about 1 cm^2^) was isolated and fixed in 4% paraformaldehyde. Three slides from each block were evaluated for the percentage of apoptotic cells using a TUNEL assay kit (Roche Diagnostics, Basel, Switzerland) according to the manufacturer's instructions. Normal myocardial nuclei were blue-green, while apoptotic nuclei were different shades of brown. Both total and TUNEL-positive myocytes were counted in each field under high magnification (×400) in five independent randomly-selected fields. Results are expressed as apoptotic index (CAI): (number of TUNEL-positive myocytes/total myocytes) ×100% [20].

### GRP78 and CHOP protein expression by immunohistochemistry

After conventional dewaxing and microwave antigen retrieval, sections were incubated with 1:200 rabbit antibodies against swine GRP78 (Santa Cruz Biotechnology, Santa Cruz, CA, USA) and CHOP (Abcam, Cambridge, MA, USA)at 4°C overnight. After washing with PBS, a biotin-labeled antibody was added for 30 min. DAB was used for coloration, and the sections were sealed. Images were collected in five randomly-selected x400 fields using a microscope equipped with a digital camera. The average optical density was measured using the CMIAS multifunctional true color pathological image analysis system (Air Force General Hospital and the Beijing University of Aeronautics and Astronautics).

### GRP78 and CHOP protein expression by western blot

Tissues of LVAW were taken from each experimental miniature swine, cut into pieces at 4°C in a mortar, homogenized, and centrifuged at 10,000 rpm at 4°C for 15 min. A BCA kit was used to quantify the total protein concentration of the supernatant. Samples were mixed with distilled water and 5× SDS-loading buffer. Proteins were separated using 10% SDS polyacrylamide gel electrophoresis. Proteins were transferred onto a nitrocellulose membrane. GRP78 and GADD153/CHOP antibodies (both 1:1000) were added and incubated at 4°C overnight. After washing the membrane, sheep anti-rabbit IgG (1:1000) was added at room temperature and incubated for 2 h. Antibodies against β-actin (Santa Cruz Biotechnology, Santa Cruz, CA, USA, 1:2000) were used for normalization. Bands were revealed using an ECL kit (Cell Signaling, Danvers, MA, USA). The image analysis software Image-J was used to analyze the protein bands for integrated optical density (IOD). IOD = average optical density value × area. Target protein IOD value/β-actin IOD ratio reflects the level of target protein expression.

### Statistical analysis

All data are expressed as means ±standard deviation. The maximal tolerated +Gz stress values between the different groups were evaluated using the Kruskal-Wallis test for nonparametric independent samples. Data before and after +Gz stress exposure were evaluated by one-way analysis of variance (ANOVA) with the least significant difference (LSD) test for post-hoc analysis. All analyses were performed using SPSS 13.0 (SPSS Inc., Chicago, Illinois, USA). P-values ≤0.05 were considered statistically significant.

## Results

### Surgery and model success

**Fig 1:**
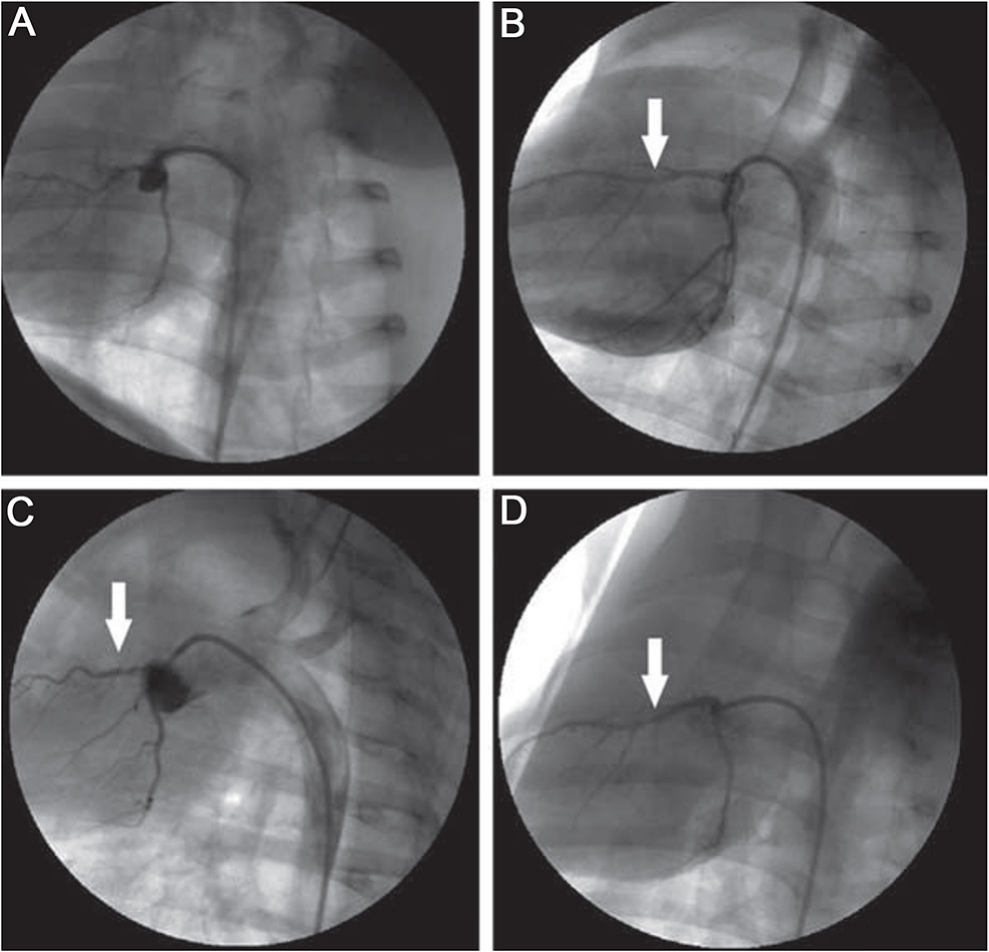
Coronary artery angiogram after left anterior descending (LAD) artery ligation. (A) Normal coronary artery in the sham-operated group. (A) Mild stenosis in LAD artery; the arrow indicates about 30% stenosis in the proximal LAD artery. (C) Moderate stenosis in the LAD artery; the arrow indicates about 60% stenosis in the proximal LAD artery. (d) Severe stenosis in the LAD artery; the arrow indicates about 80% stenosis in the proximal LAD artery. Arrows show the location of stenosis.

Twenty miniature swine underwent LAD artery ligation and the degree of CAS was quantified by QCA. Five swine underwent sham surgery (Fig 1A). Seven swine were in the mild stenosis group (36.8±7.4% stenosis; Fig 1B), six were in the moderate stenosis group (61.5±6.2% stenosis; Fig 1C), and seven were in the severe stenosis group (81.9±8.6% stenosis; Fig 1D). The reference vascular diameter was 2.89±0.28 mm. Two swine in the severe stenosis group suffered from ventricular fibrillation, recovered for a brief time after acute treatment, but were euthanized 8 and 48 h after surgery because they developed loss of appetite, forelimb weakness, hypotonia and unresponsiveness to external stimuli. Pathological analysis showed that the anterior wall of the left ventricle was dark red, which was consistent with the pathological features of acute myocardial infarction. Light microscopy revealed coagulation necrosis of myocardial cells, nuclear fragmentation, increased cell gap, intercellular edema and neutrophil infiltration. Apart from these, the ST-T wave in the precordial V1-4 ECG leads showed significant arched elevation and finally formed a pathological Q wave, in accordance with acute anterior myocardial infarction.Therefore, the success rate of the surgery was 100%, and the success rate of the model was 90%.

### Maximal tolerated +Gz stress decreased with increasing stenosis

**Fig 2:**
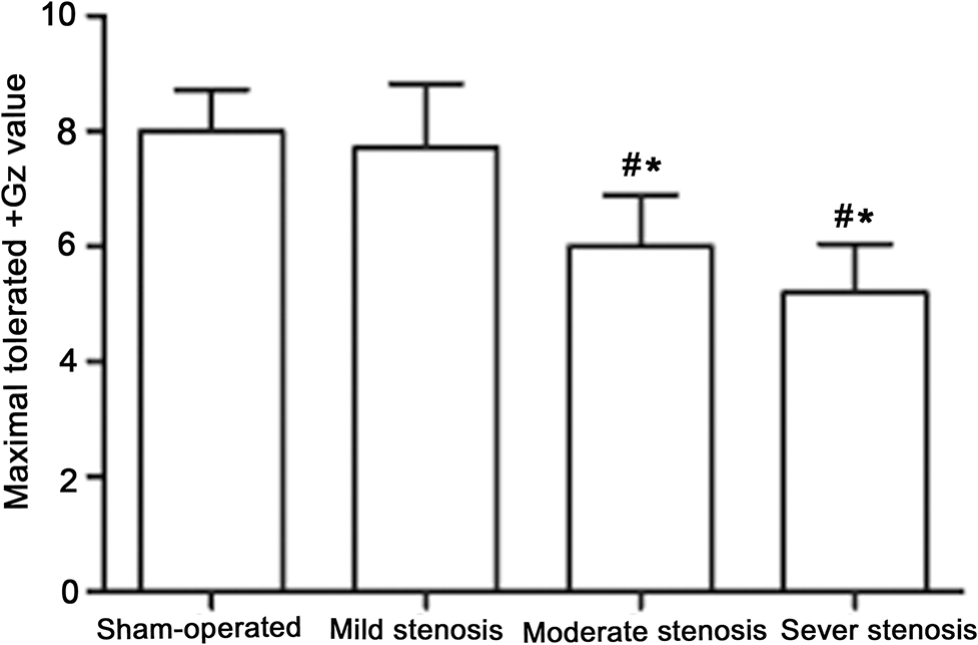
Maximal tolerated +Gz values in different stenosis groups. (n = 5 for the sham-operated group; n = 5 for the severe stenosis group; n = 6 for the moderate group and n = 7 for the mild stenosis group). *P<0.05 vs. the sham-operated group, #P<0.05 vs. the mild stenosis group.

A total of 23 small swine underwent the centrifuge experiment. The +Gz stress was considered as the maximum tolerated +Gz stress when ECG monitoring displayed risk signals. The experimental results showed that the maximum tolerated +Gz stress values were: +8.00±0.71 Gz in the sham-operated group, +7.71±1.11 Gz in the mild stenosis group, +6.00±0.89 Gz in the moderate stenosis group (P<0.05 vs. the sham-operated and mild stenosis groups), and+5.20±0.84 Gz in the severe stenosis group (P<0.05 vs. the sham-operated and mild stenosis groups) (Fig 2). There were no differences between the sham-operated and the mild stenosis groups, nor between the moderate and severe stenosis groups.

### Myocardial histopathological changes after +Gz stress

**Fig 3:**
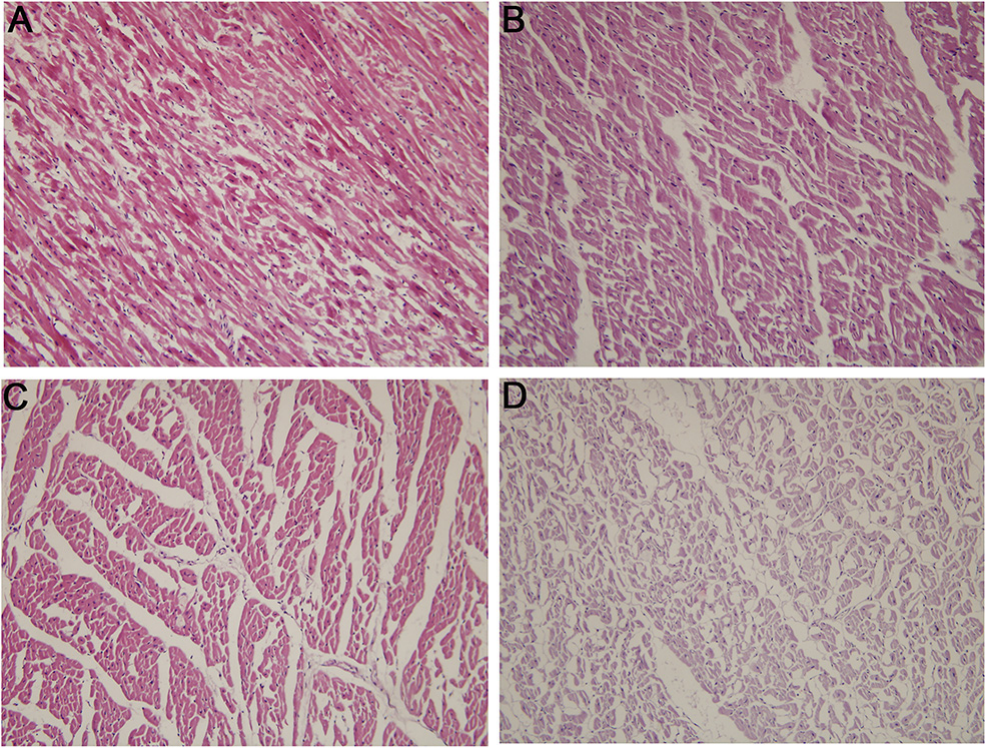
Pathological changes in myocardial tissue (H&E, ×200). (A) Sham-operated group. (B) Mild stenosis group. (C) Moderate stenosis group. (D) Severe stenosis group.

Myocardial histopathological changes were evaluated by light microscopy.In the sham-operated group,myocardial cells were arranged regularly, boundary and texture were clear, endochylema was stained uniformly, full dark blue nuclei were visible, and there was no interstitial edema. In the mild stenosis group, myocardial cells showed minor deformation, interspace was slightly increased, endochylema was generally uniformly stained, and there was slight interstitial edema. In the moderate stenosis group, myocardial cell showed an irregular arrangement and some were broken, interspace was broad, and the extent of myocardial injury was between that of the mild and severe groups. In the severe stenosis group, myocardial cells were obviously deformed, were disordered, boundaries were not clear, and there were significant gaps between cells (Fig 3A–3D).

### Effect of +Gz stress on myocardial cell apoptosis of miniature swine model

**Fig 4:**
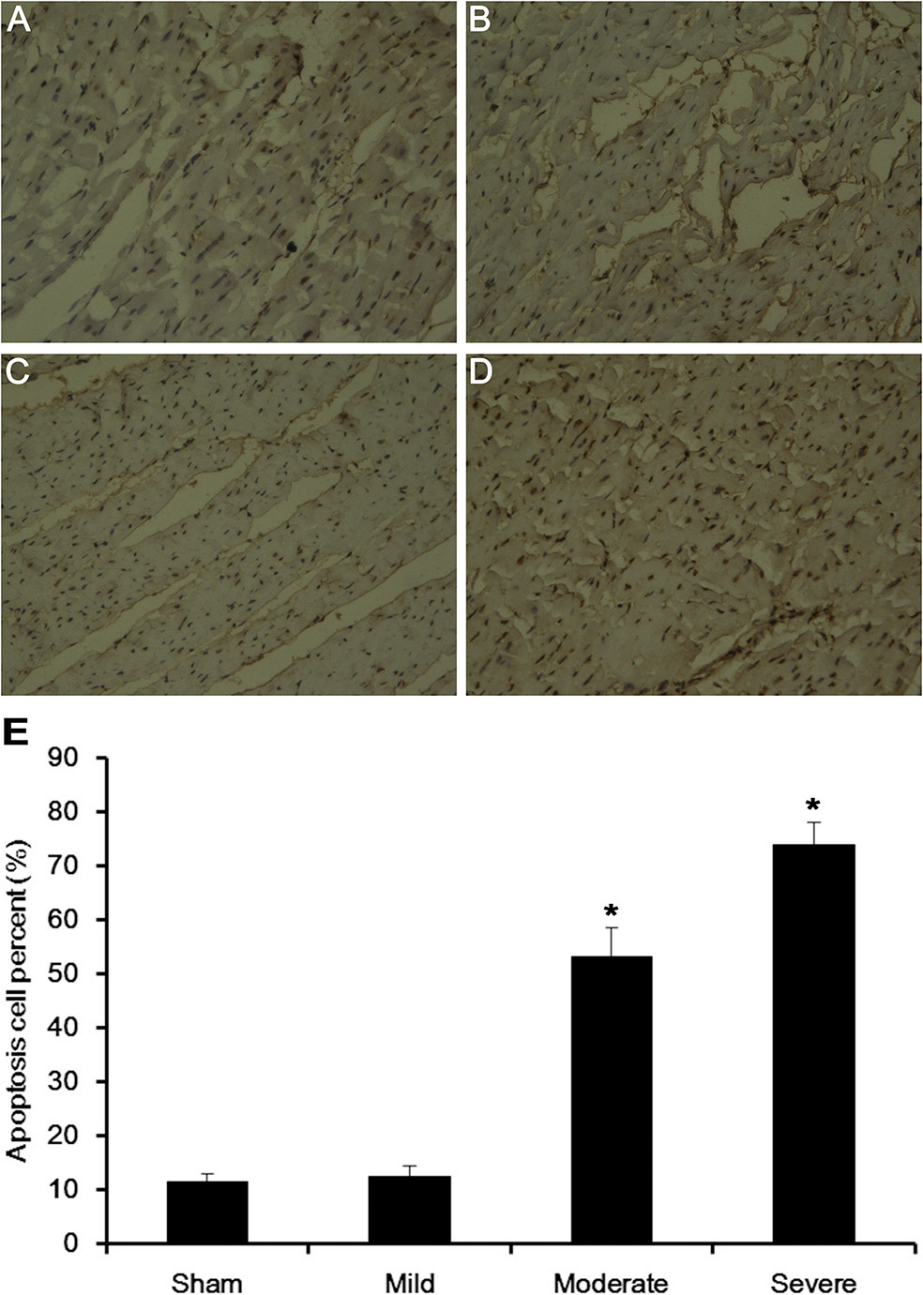
Myocardial cell apoptosis of each group (DAB coloration, ×400). (A) Sham group. (B) Mild stenosis group. (C) Moderate stenosis group. (D) Severe stenosis group. (E) Quantification of cell apoptosis in the four stenosis groups. *P<0.05 vs. the sham operated group.

After maximum +Gz stress, there was a small amount of apoptotic cells in the sham-operated and mild stenosis groups. Myocardial apoptosis rate (shown as apoptotic index in TUNEL assays) was 12.16±1.25% and 13.32±1.45%, respectively (P>0.05). In the moderate stenosis group, the myocardial cell apoptosis rate was 43.53±8.42% (P<0.05 vs. the sham-operated group). In the severe stenosis group, the number of apoptosis cells was higher (60.50+9.35%, P<0.05vs. the sham-operated group) (Fig 4E). Representative TUNEL assay pictures are shown in Fig 4.

### Effect of +Gz stress on MDA content and SOD activity of myocardial tissue

As shown in Table 1, compared with the sham-operated group, the MDA content of myocardial tissue in the mild stenosis group was increased slightly, the SOD activity was decreased slightly, but without significant difference (P>0.05). As the degree of CAS increased, the MDA content of myocardial tissue increased, and SOD activity decreased. The MDA content in the moderate and severe stenosis groups was increased 1.89- and 4.91-folds compared with the sham-operated group (both P<0.05). The SOD activity in the moderate and severe stenosis groups was 86.44% and 78.29% of that of the sham-operated group (both P<0.05).

**Table 1:** Changes in MDA content and SOD activity in myocardial tissue.

Group	Sham (n = 5)	Mild stenosis (n = 7)	Moderate stenosis (n = 6)	Severe stenosis (n = 5)
MDA(nmol/mg prot)	1.46±0.13	1.55±0.11	2.76±0.25*	7.17±0.33*
SOD (U/mg prot)	235.64±4.01	226.59±10.38	203.68±4.86*	184.48±8.04*

*P<0.05 vs. the sham group.

Results are presented as mean ± SD.

All swine were exposed to +Gz.

### Changes in GRP78 and CHOP protein expressions in myocardial tissue after +Gz stress by immunohistochemistry

**Fig 5:**
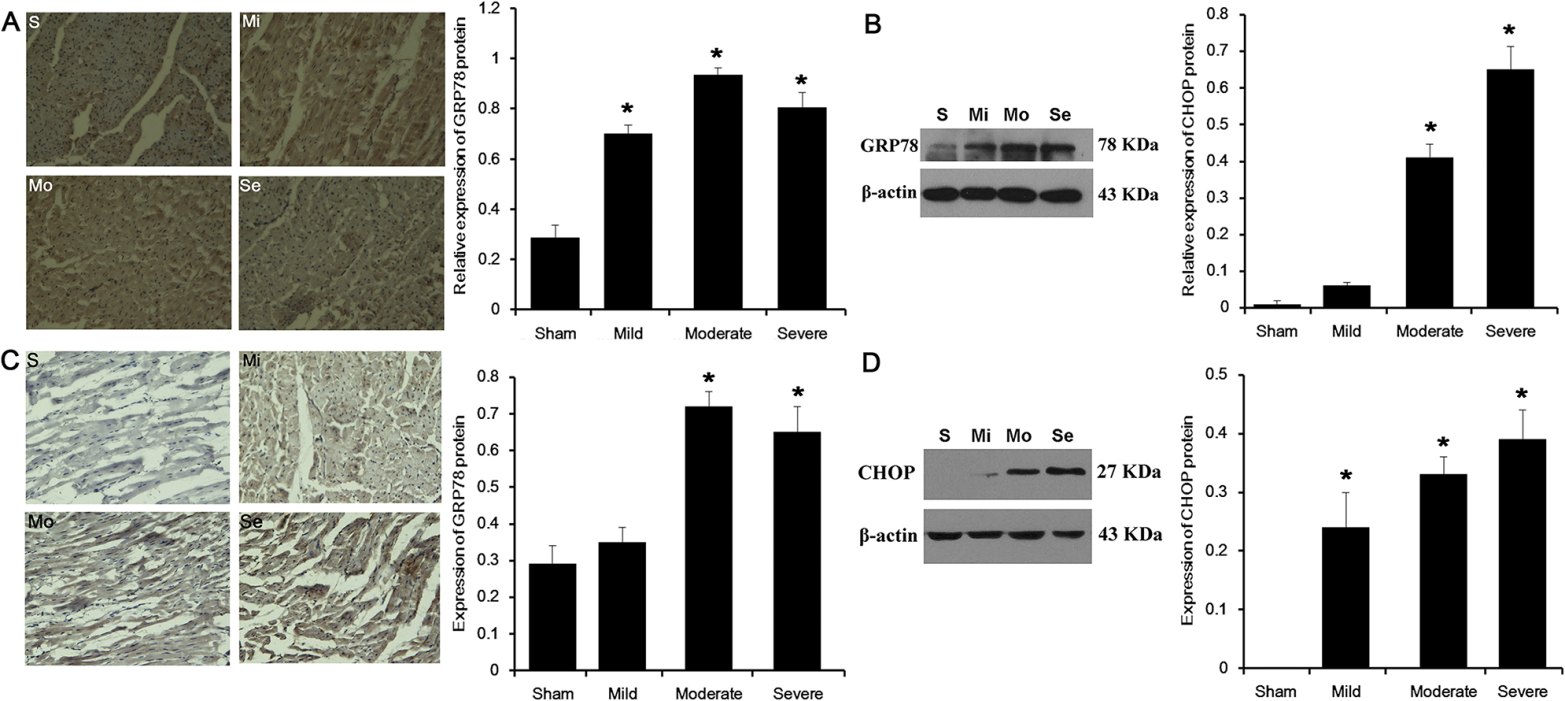
Changes in GRP78 and CHOP protein expressions in myocardial tissue after +Gz stress. (A) GRP78 immunohistochemistry in myocardial cells in each group (×400). (B) Effects of + Gz stress on GRP78 protein expression (western blot) in each group. (C) CHOP immunohistochemistry in myocardial cells of each group (x400). (D) Effect of + Gz stress on CHOP protein expression (western blot) in each group. S: sham-operated group; Mi: mild stenosis group; Mo: moderate stenosis group; Se: severe stenosis group. *P<0.05 vs. the sham operated group.

The expression of GRP78 was located in the myocardial cell cytoplasm (Fig 5A). GRP78 was weakly positive in the sham-operated and mild stenosis groups, with average optical density values of0.29±0.05 (n = 5) and0.35±0.04 (n = 7), respectively (P>0.05). GRP78 was strongly positive in the moderate stenosis group, with an average optical density value of0.72±0.04 (n = 6)(P<0.05 vs. the sham-operated group). GRP78 was also strongly positive in the severe stenosis group, but was slightly lower than in the moderate stenosis group, with an average optical density value of 0.65±0.07 (n = 5) (P<0.05 vs. the sham-operated group).

The expression of CHOP was located in the myocardial cell cytoplasm (Fig 5C). CHOP was absent in the sham-operated animals. CHOP was weakly positive in the mild stenosis groups, with an average optical density value of0.24±0.06 (n = 7). CHOP was positive in the moderate stenosis group, with an average optical density value of0.33±0.03 (n = 6) (P<0.05 vs. the mild stenosis group). CHOP was strongly positive in the severe stenosis group, with an average optical density value of 0.39±0.05 (n = 5) (P<0.05 vs. the mild stenosis group).

### Changes in GRP78 and CHOP protein expressions in myocardial tissue after +Gz stress by western blot

Western blot (Fig 5B) showed GRP78 expression levels of 0.29±0.05, 0.70±0.04, 0.93±0.03 and 0.80±0.06 in the sham-operated, mild stenosis, moderate stenosis and severe stenosis groups, respectively (all stenosis groups: P<0.05 vs. the sham-operated group).

Using western blot (Fig 5D), CHOP expression was negative in the sham-operated group, and positive in the stenosis groups, with average values of 0.06±0.01, 0.41±0.04 and0.65±0.06 in the mild, moderate and severe stenosis groups, respectively (moderate and severe stenosis groups: P<0.05 vs. the mild stenosis group).

## Discussion

The aim of the present study was to investigate the effects of myocardial injury in a minimally-invasive miniature swine model with different levels of CAS and exposed to maximal tolerated +Gz. Results showed that compared with the sham-operated group, the maximal tolerated +Gz values of the moderate stenosis and severe stenosis groups were decreased. Compared with sham animals, after exposure to maximum +Gz, apoptotic cells of the moderate and severe stenosis group were increased, MDA content was increased, and SOD activity was reduced. After exposured to maximum +Gz, GRP78 protein expression was low in the sham-operated and mild stenosis groups, while expression was high in the moderate and severe stenosis groups. CHOP protein expression was not observed in the sham-operated group, while expression was high in the moderate and severe stenosis groups.

MDA is a cytotoxic metabolite of the oxidation of membrane unsaturated fatty acids, and its levels represent the degree of oxidative cell damage. SOD is a superoxide anion radical-scavenging enzyme able to remove oxygen free radicals, and its activity indirectly reflects the antioxidant status. Through observing the changes in MDA and SOD levels, we can grasp an idea of the dynamic process of free radicals [21].Myocardial ischemia reperfusion and high +Gz stress can lead to abnormal myocardial free radical metabolism, which affect the structure and function of the heart, may increase membrane lipid peroxidation, may cause cell calcium overload and outflow, and may induce mitochondrial damage. Results showed that the mild stenosis group was characterized by a mild elevation of MDA levels and a slight decline in SOD. These changes were more important with the severity of CAS. After +Gz stress, the oxidative stress response was significantly increased in moderate and severe coronary stenosis models, due to a decreased ability to remove ROS, leading to increased cell damage. Therefore, this increased state of oxidative stress produced in different coronary stenosis models under the maximum tolerated +Gz stress may cause some damage to the body. These results are comparable with results obtained in the brains of rats exposed to repeated +Gz exposure [22].

ER is responsible for protein biosynthesis, correct folding, and post-translational modifications of secretory and membrane proteins. However, under physiological or pathological stress, an accumulation of misfolded and unfolded proteins in ER lumen results in ERS [23,24]. A study has shown that I/R can lead to a serious imbalance of myocardial ER functions, and that inhibiting oxidative stress is one of the important mechanisms to protect the function of endoplasmic reticulum and relieve ERS [25]. GRP78 reflects the activation of ERS [26]. At the same time, over-expression of GRP78 can enhance the capacity of ER handling of unfolded proteins, and reflects the adjustment ability of ERS. The present study observed that the expression of GRP78 in groups with different CAS degrees and submitted to maximum tolerated +Gz stress was increased with increasing degree of stenosis, suggesting that +Gz stress may cause an ERS response in ischemic myocardium.

CHOP is a member of the C/EBP transcription factor family that heterodimerizes with other C/EBPs and mediates the induction of apoptosis through the mitochondrial pathway. It was found that enforced dimerization of Bax, a pro-apoptotic Bcl-2 family member, and consequent translocation of Bax-dimers from the cytosol to mitochondria play a key role in ERS-mediated apoptosis downstream of CHOP induction [27]. Szegezdiet al. [28] have found that CHOP act as a common downstream apoptotic signaling molecules in multiple apoptosis signaling pathways mediated by ERS, and that CHOP protein expression reflects the degree of apoptosis mediated by ERS. The present study observed that under the maximum tolerated +Gz stress, CHOP was not expressed in the sham-operated group, and weakly positive in the mild stenosis group; however, as the extent of CAS was aggravating, the expression of CHOP gradually increased. The trend in CHOP expression followed the trend in apoptosis observed using the TUNEL method, which is supported by a previous study in brains of rats [29]. Therefore, increased extent of coronary stenosis can gradually activate the apoptosis response mediated by ERS. Then, ERS apoptosis response may be regulated by the degree of coronary artery stenosis. Results also showed that the GRP78 expression in the moderate stenosis group was the highest, but that CHOP protein expression was the highest in the severe stenosis group, which suggests that CHOP expression is affected by GRP78 [30]. However, in the severe stenosis group, GRP78 and CHOP expressions showed inconsistencies, suggesting that other pathways may be involved in the response to +Gz in animals with severe CAS [30].

The results showed that with increasing degree of CAS, the maximum tolerated +Gz acceleration value was reduced, and that the degree of oxidative stress, cell apoptosis and ERS were increased. These changes may be related to both CAS degree and +Gz acceleration. Both CAS and +Gz stress can induce cell apoptosis through ERS, increasing myocardial ischemia and myocardial remodeling, which is supported by a previous studies showing changes in muscle fiber after +Gz [31], as well as a study showing changes in cardiomyocytes and mitochondria damage after +Gz [32]. This study also found that animals with mild coronary stenosis could tolerate the same maximum +Gz acceleration than sham-operated animals (without CAS). In addition, ERS and oxidative stress were comparable in these animals, which may be due to the homeostatic regulation of ERS in myocardial tissue [30].

However, the exact mechanisms leading to ERS after +Gz remain elusive and further study is necessary to elucidate them in animals. Some studies performed in eukaryote plant cells might suggest that disruption of the actin cytoskeleton and movements of the organelles within the cells by acceleration forces may be involved in ERS [33]. A study in rats suggests that cardiomyocytes of rats exposed at +10 Gz for 5 min showed damaged mitochondrial ultrastructure, impaired mitochondrial respiratory chain, decreased content of antioxidant proteins, and increased oxidative stress [34]. A study in rats suggested that hypergravity influenced the development of the brain through increased oxidative stress on Purkinje cells [35]. Rats exposed to short (30 sec) +10 Gz stress showed increased brain lipid peroxidation [36]. Since oxidative stress is involved in ERS [24,37], +Gz may affect the ER through increased oxidative stress, but studies are required to address this specific issue since none focused specifically on the effects of +Gz on ERS.

The present study is not without limitation. The need for surgery and handling of animals in the centrifuge increased the cost of the study, limiting the number of animals in each group and limiting result refinement. Second, myocardial tissue cannot be sampled in the same animals before +Gz stress, preventing the assessment of the exact mechanisms involved. Finally, we did not have a control group made of animals with stenosis, but that were not exposed to +Gz. In future studies, we will aim at mild and moderate coronary artery stenosis models exposed to different gradients of +Gz acceleration, in order to study the role of apoptosis induced by +Gz stress acceleration in ERS.

## Conclusions

Under maximum exposure to +Gz stress, different levels of CAS led to different levels of myocardial injury. ER response is involved in the apoptosis of cardiomyocytes after +Gz stress.

## Supporting Information

S1 Raw Data (DOCX)
