# Supplementary material for: Endoplasmic Reticulum Is Involved in Myocardial Injury in a Miniature Swine Model of Coronary Artery Stenosis Exposed to Acceleration-Associated Stress
Source: PLoS One. 2015 Jul 13;10(7):e0132654. doi: 10.1371/journal.pone.0132654 (PMC4500442; doi:10.1371/journal.pone.0132654)
Supplement: S1 Raw Data — (DOCX) [file pone.0132654.s001.docx]

**Figure 2.** Maximal tolerated +Gz value in different stenosis group (n=5 for the sham-operated and severe stenosis groups; n=6 for the moderate and n=7 forthe mild stenosis groups). *P<0.05 vs. the sham-operated group.

| G | sham | mild | moderate | severe |
| --- | --- | --- | --- | --- |
| 1 | 7 | 8 | 7 | 5 |
| 2 | 9 | 7 | 5 | 6 |
| 3 | 8 | 7 | 7 | 6 |
| 4 | 8 | 9 | 5 | 4 |
| 5 | 8 | 9 | 6 | 5 |
| 6 |  | 8 | 6 |  |
| 7 |  | 6 |  |  |
| 平均 | 8.00±0.71 | 7.71±1.11 | 6.00±0.89 | 5.20±0.84 |

**凋亡指数（单位：%）**

**Figure 4.** 凋亡：

| 凋亡 | sham | mild | moderate | severe |
| --- | --- | --- | --- | --- |
|  | after+Gz | after+Gz | after+Gz | after+Gz |
| 1 | 10.38 | 15.67 | 46.78 | 70.23 |
| 2 | 12.79 | 9.89 | 56.46 | 69.87 |
| 3 | 9.87 | 12.75 | 60.58 | 75.65 |
| 4 | 11.46 | 10.76 | 48.56 | 72.56 |
| 5 | 13.23 | 10.45 | 55.78 | 80.79 |
| 6 |  | 12.85 | 50.94 |  |
| 7 |  | 14.23 |  |  |
| 平均 |  |  |  |  |

**Figure 4.** MDA含量：

| MDA | sham | mild | moderate | severe |
| --- | --- | --- | --- | --- |
|  | after+Gz | after+Gz | after+Gz | after+Gz |
| 1 | 1.5957 | 1.5532 | 2.4043 | 7.5745 |
| 2 | 1.4468 | 1.6170 | 2.5745 | 7.3191 |
| 3 | 1.3404 | 1.4042 | 2.8425 | 6.9876 |
| 4 | 1.3308 | 1.7235 | 2.9753 | 6.7235 |
| 5 | 1.6075 | 1.4654 | 3.0787 | 7.2365 |
| 6 |  | 1.6342 | 2.6875 |  |
| 7 |  | 1.4785 |  |  |
| 平均 |  |  |  |  |

**Figure 4.** SOD 含量：

| SOD | sham | mild | moderate | severe |
| --- | --- | --- | --- | --- |
|  | after+Gz | after+Gz | after+Gz | after+Gz |
| 1 | 238.90 | 221.87 | 208.03 | 189.69 |
| 2 | 231.76 | 224.62 | 206.38 | 193.79 |
| 3 | 233.96 | 229.56 | 204.74 | 179.24 |
| 4 | 240.87 | 206.89 | 198.89 | 185.98 |
| 5 | 232.74 | 232.65 | 207.63 | 173.74 |
| 6 |  | 230.98 | 196.46 |  |
| 7 |  | 239.54 |  |  |
| 平均 |  |  |  |  |

**Figure 4.** CHOP含量（western）：

| GRP-78 | sham | mild | moderate | severe |
| --- | --- | --- | --- | --- |
|  | after+Gz | after+Gz | after+Gz | after+Gz |
| 1 | 0.02 | 0.05 | 0.38 | 0.7 |
| 2 | 0.00 | 0.07 | 0.45 | 0.67 |
| 3 | 0.01 | 0.06 | 0.40 | 0.58 |
| 4 |  |  |  |  |
| 5 |  |  |  |  |
| 6 |  |  |  |  |
| 7 |  |  |  |  |
| 平均 |  |  |  |  |

**Figure 4.** GRP -78含量（western）：

| GRP-78 | sham | mild | moderate | severe |
| --- | --- | --- | --- | --- |
|  | after+Gz | after+Gz | after+Gz | after+Gz |
| 1 | 0.24 | 0.67 | 0.90 | 0.74 |
| 2 | 0.28 | 0.69 | 0.94 | 0.81 |
| 3 | 0.34 | 0.74 | 0.96 | 0.86 |
| 4 |  |  |  |  |
| 5 |  |  |  |  |
| 6 |  |  |  |  |
| 7 |  |  |  |  |
| 平均 |  |  |  |  |

**Figure 4.** GRP -78含量（免疫组化）：

| GRP-78 | Sham（n=5） | Mild(n=7) | Moderate(n=6) | Severe(n=5) |
| --- | --- | --- | --- | --- |
|  | after+Gz | after+Gz | after+Gz | after+Gz |
| 1 | 0.26 | 0.38 | 0.73 | 0.62 |
| 2 | 0.24 | 0.28 | 0.71 | 0.65 |
| 3 | 0.36 | 0.34 | 0.77 | 0.75 |
| 4 | 0.27 | 0.32 | 0.66 | 0.57 |
| 5 | 0.34 | 0.37 | 0.71 | 0.66 |
| 6 |  | 0.42 | 0.72 |  |
| 7 |  | 0.36 |  |  |
| 平均 | 0.29±0.05 | 0.35±0.04 | 0.72±0.04 | 0.65±0.07 |

**Figure 5.** CHOP含量（免疫组化）：

| GRP-78 | sham | mild | moderate | severe |
| --- | --- | --- | --- | --- |
|  | after+Gz | after+Gz | after+Gz | after+Gz |
| 1 |  | 0.35 | 0.32 | 0.46 |
| 2 |  | 0.23 | 0.37 | 0.35 |
| 3 |  | 0.28 | 0.30 | 0.39 |
| 4 |  | 0.18 | 0.28 | 0.38 |
| 5 |  | 0.19 | 0.36 | 0.35 |
| 6 |  | 0.22 | 0.34 |  |
| 7 |  | 0.24 |  |  |
| 平均 | 无表达 | 0.24±0.06 | 0.33±0.03 | 0.39±0.05 |
